# Seroconversión inducida por vacuna experimental al VIH: un reto clínico fuera de los ensayos clínicos

**DOI:** 10.1016/j.aprim.2025.103247

**Published:** 2025-04-21

**Authors:** Sergio Ferra Murcia, Marta Segura Díaz, Antonio Ramón Collado Romacho

**Affiliations:** a Unidad de Gestión Clínica de Enfermedades Infecciosas, Hospital Universitario Torrecárdenas, Almería, Andalucía, España; b Medicina Interna, Hospital Universitario Torrecárdenas, Almería, Andalucía, España

La infección por el virus de la inmunodeficiencia humana (VIH) sigue siendo una entidad prevalente e incidente tal y como reflejan los últimos datos disponibles del programa conjunto de las naciones unidas sobre el VIH (ONUSIDA), donde, al cierre del año 2023, 1,3 millones de personas contrajeron el VIH; 39,9 millones estaban viviendo con el VIH y 630.000 personas murieron por enfermedades relacionadas con el síndrome de inmunodeficiencia adquirida (sida)^1^.

La persistencia de primoinfecciones por el VIH, las infecciones ocultas asintomáticas y los pacientes que no reciben tratamiento o lo realizan de una forma inadecuada mantienen la epidemia de VIH de forma activa^2^.

Es por ello, por lo que las autoridades sanitarias han establecido objetivos específicos dirigidos a disminuir los nuevos casos de infección, motivando un diagnóstico precoz y favoreciendo un mayor acceso a los tratamientos antirretrovirales (TAR) de forma global. En este escenario es de vital importancia una adecuada coordinación y colaboración, en el manejo compartido de los pacientes que viven con VIH, entre Medicina Familiar y Comunitaria y las consultas de Enfermedades Infecciosas en el entorno hospitalario. Ejemplo de ello es la guía conjunta elaborada por el panel de expertos del grupo de estudio del sida (GeSIDA) y de la Sociedad Española de Medicina de Familiar y Comunitaria (semFYC) presentada en 2022, consolidando los conocimientos y evidencias actuales en busca de aunar esfuerzos que impacten en la consecución de estos objetivos compartidos^3^.

La evolución de las técnicas de diagnóstico disponibles ha permitido afrontar recomendaciones universales para la búsqueda activa de casos y tratar de reducir la infección oculta, con el empleo de técnicas con distinta base antigénica y serologías de cuarta generación que permiten reducir los periodos ventana a unos 13-15 días, gracias a la incorporación de la detección del antígeno p24. Los test confirmatorios como el *«Western Blot»* (WB) y la detección del genoma del VIH permiten complementar el diagnóstico en situaciones complejas^4^.

El diagnóstico precoz es un eje sobre el que pivota este abordaje conjunto y multidisciplinar, pero «en vida real» podemos encontrar alguna situación novedosa, inesperada y de difícil manejo, como la acontecida en el presente caso que aquí documentamos. Se trata de un varón de 35 años de edad, con múltiples parejas sexuales y relaciones esporádicas de riesgo no protegidas. Itinerante en varias ciudades de la geografía española, consulta en su nuevo centro de salud para realizarse un control serológico y cribado de infecciones de transmisión sexual (ITS). Entre ellas, se solicita una serología VIH (ELISA de cuarta generación) que resulta positiva, a lo que se añade otra técnica con distinta base (quimioluminiscencia) que también resulta positiva con test confirmatorio (WB) negativo. Se repite todo al mes, con los mismos resultados, por lo que se determina carga viral VIH en plasma que también es negativa. ¿Estamos por tanto ante un falso positivo? Dos determinaciones separadas un mes con el mismo resultado y exposiciones de riesgo ¿es un periodo ventana?, ¿es un controlador de élite?

Para responder a estas preguntas, una vez más, necesitamos de una adecuada anamnesis: este paciente había sido participante en su itinerancia geográfica de un ensayo clínico donde había recibido una vacuna experimental frente al VIH, motivando una seropositividad de los test diagnósticos disponibles, entidad conocida como *«vaccine-induced seropositive/reactivity: VISP/R»*^5^.

Esta situación podemos encontrarla en vida real, en el devenir de las consultas y por tanto debemos conocerla y manejarla. Si bien la producción de anticuerpos tras haber recibido una vacuna experimental es un hecho «deseable», carecemos de guías de práctica clínica que recojan la forma de proceder adecuada con estos pacientes.

## Financiación

Confirmamos que la elaboración de este manuscrito no ha sido financiada por ninguna entidad ni institución.

## Consideraciones éticas

El paciente ha otorgado su consentimiento, habiéndose cumplido la normativa vigente.

## Conflicto de intereses

Los autores declaran no tener ningún conflicto de intereses.
